# Apps in der Rheumatologie: Bedarf es einer App in der Therapie der axialen Spondyloarthritis?

**DOI:** 10.1007/s00393-021-01104-1

**Published:** 2021-10-07

**Authors:** Patrick-Pascal Strunz, Maxime Le Maire, Tobias Heusinger, Ludwig Hammel, Michael Gernert, Eva C. Schwaneck, Johanna Callhoff, Jan Portegys, Marc Schmalzing, Hans-Peter Tony, Matthias Froehlich

**Affiliations:** 1 Medizinische Klinik II, Abteilung für Rheumatologie und klinische Immunologie, Universitätsklinikum Würzburg, Oberdürrbacher Straße 6, 97080 Würzburg, Deutschland; 2 Medizinische Fakultät, Universität Würzburg, Würzburg, Deutschland; 3 Deutsche Vereinigung Morbus Bechterew e. V., Schweinfurt, Deutschland; 4 Asklepios Klinik Altona, Hamburg, Deutschland; 5 Epidemiologie, Deutsches Rheuma-Forschungszentrum Berlin (DRFZ), Berlin, Deutschland; 6 Institut für Sozialmedizin, Epidemiologie und Gesundheitsökonomie, Charité – Universitätsmedizin Berlin, Berlin, Deutschland

**Keywords:** Morbus Bechterew, DiGA, Bewegungstherapie, Physiotherapie, Trainingsprogramm, Bechterew’s disease, Digitalization in medicine, Exercise program, Physiotherapy, Training programm

## Abstract

**Hintergrund:**

Digitale Gesundheitsanwendungen (DiGA) halten in vielen Bereichen der Medizin Einzug und haben das Potenzial, die Patientenversorgung zu revolutionieren. In der Rheumatologie wäre der Einsatz bei der axialen Spondyloarthritis (axSpA) in Form einer Trainings- und Bewegungs-App denkbar. In einer repräsentativen Umfrage unter Patienten mit axSpA sollte daher ermittelt werden, ob eine solche App aktuell benötigt wird.

**Methodik:**

Durchführung einer anonymen Onlinebefragung bei axSpA-Patienten der Deutschen Vereinigung Morbus Bechterew e. V. mittels eines Fragebogens; Datenauswertung mittels Excel und GraphPad Prism.

**Ergebnisse:**

Es nahmen 435 axSpA-Patienten an der Befragung teil. Von den Befragten sehen 84 % die Entwicklung einer speziell auf axSpA abgestimmten Bewegungs-App als notwendig an und genauso viele wollen diese auch nutzen. Patienten unter 60 Jahre, Patienten unter 60-Jahre mit Biologika- oder Januskinase-Inhibitor-Therapie und Patienten mit häufigen Rückenschmerzen geben im Vergleich zur jeweiligen Kontrollsubgruppe einen höheren Bedarf an (jeweils *p* < 0,001).

**Schlussfolgerung:**

Die Entwicklung einer Bewegungs-App für die axSpA wird von einem Großteil der Betroffenen als notwendig angesehen, wobei jüngere und intensiver medikamentös therapierte Patienten einen höheren Bedarf zu haben scheinen.

**Zusatzmaterial online:**

Die Online-Version dieses Beitrags (10.1007/s00393-021-01104-1) enthält den Studienfragebogen.

## Abstract (en)

**Background:**

Digital health applications/apps (DiGA) are entering many medical disciplines and have the potential to revolutionize patient care. In rheumatology, the use for axial spondyloarthritis (axSpA) would be conceivable in the form of an exercise app. Therefore, a representative survey among axSpA patients was conducted to determine the need for an axSpA exercise app.

**Materials and methods:**

An anonymous online survey among axSpA patients of the German Bechterew’s Disease Association was conducted using a questionnaire; data were analysed using Excel, and GraphPad Prism.

**Results:**

Four hundred and thirty-five axSpA patients participated in the survey. Eighty-four percent of the participants responded that there is a need to develop an axSpA-specific exercise app, and the same proportion want to use it. Patients under 60 years, patients under 60 years on biologics or Janus kinase inhibitor therapy, and patients with frequent back pain reported a greater need than their respective control subgroups (*p* < 0.001 in each case).

**Conclusion:**

The development of an exercise app for axSpA is considered necessary by a large proportion of the patients; younger and more intensively treated patients appear to have a greater need.

Im Zeitalter der Digitalisierung haben medizinische Apps Einzug in die Patientenversorgung gehalten. Während digitale Gesundheitsanwendungen (DiGA) bereits in der Neurologie, Psychiatrie und Onkologie eingesetzt werden und verordnungsfähig sind, gibt es noch keine Untersuchungen, ob auch für rheumatische Erkrankungen ein Bedarf besteht. Dies wurde daher anhand der axialen Spondyloarthritis mittels einer Umfrage in einer Selbsthilfegruppe untersucht. In diesem Beitrag werden die Ergebnisse dieser Umfrage vorgestellt.

## Hintergrund/Fragestellung

Bei kaum einer Erkrankung des rheumatischen Formenkreises spielen Bewegungstherapie und physikalische Beübung eine so große Rolle wie in der Therapie der axialen Spondyloarthritis (axSpA) [1]. In jüngsten Studien konnte gezeigt werden, dass intensives Training nicht nur einen positiven Effekt auf die Ankylosierung der Wirbelsäule hat, sondern sogar direkt positiv modulierend auf den inflammatorischen Prozess selbst wirken kann [2]. Nach dem korrekten Erlernen von speziellen Übungen in physiotherapeutischen Einrichtungen, Fitnessstudios oder Bechterew-Therapiegruppen spielt die Fortsetzung der Beübung der Wirbelsäule im häuslichen Umfeld in Eigenregie eine wichtige Rolle [1, 3]. An dieser Stelle ist denkbar, dass digitale Gesundheitsanwendungen (DiGA), die jüngst in vielen Bereichen der Medizin Einzug gefunden haben [4], einen wichtigen Beitrag zur Trainingsmotivation des Patienten und dessen Therapieadhärenz in Zukunft leisten können. Aktuell gibt es im deutschsprachigen Raum noch keine als Medizinprodukt zugelassene axSpA-Bewegungs‑/Trainings-App, weshalb die Frage interessant ist, ob es einen Bedarf für die Entwicklung einer solchen App gibt. Zur Beantwortung dieser Frage erfolgte eine anonyme Befragung von axSpA-Patienten innerhalb der Deutschen Vereinigung Morbus Bechterew e. V. (DVMB).

## Methodik

### Studiendesign

Es erfolgte die Durchführung einer anonymen, freiwilligen Onlinebefragung unter den Mitgliedern der Deutschen Vereinigung Morbus Bechterew e. V. (DVMB) via Google-Formular vom 12.02.2021 bis einschließlich 31.05.2021. Mitglieder wurden per E‑Mail oder per Post zur Befragung durch die DVMB eingeladen und willigten bei Umfragebeginn in die Durchführung der Umfrage ein. Die Daten wurden anonym gemäß DGVO zur Auswertung an das Universitätsklinikum Würzburg weitergeleitet. Da es sich um eine vereinsinterne Umfrage handelt und nicht um eine öffentliche Umfrage unter Patienten ist keine Zustimmung einer Ethik-Kommission notwendig. Die Veröffentlichung der Daten erfolgt mit Zustimmung der DVMB.

### Patientenkollektiv der DVMB

Nach Daten der DVMB aus einer anderen aktuellen Umfrage waren die Mitglieder zum Untersuchungszeitpunkt zu 50,8 % weiblich, zu 49,1 % männlich und zu 0,1 % divers. Der Altersmedian lag bei 56 Jahren (Interquartilsabstand 21 Jahre, gesamte Verteilung 12–91 Jahre). Weiter waren 74,3 % HLA-B27 positiv, 19,9 % HLA-B27 negativ, 3,0 % wurden nach eigenen Angaben darauf nicht getestet und 2,8 % konnten sich nicht mehr an den Befund erinnern. Im Jahr 2020 mussten sich 13,0 % einmalig wegen Akutbeschwerden/Schüben vonseiten der axSpA zusätzlich zur Kontrolluntersuchung beim behandelnden Rheumatologen vorstellen, 9,6 % zweimalig, 6,1 % sogar dreimalig oder häufiger und 71,3 % hatten keine Schübe oder Akutbeschwerden.

### Verwendung von Daten der Kerndokumentation und Proclair-Studie

Da die Datenerhebung mittels eines Onlinefragebogens unter Laien erfolgte, konnten keine klinischen Aktivitätsparameter (ASDAS-CRP und BASDAI) erhoben werden [1]. Daher wurde eine vergleichbare Stichprobe auf Basis der Daten der DVMB-Mitglieder aus den Registern der Kerndokumentation und der Proclair-Studie gezogen und die dokumentierten Aktivitätsparameter BASDAI und ASDAS-CRP dieser Stichprobe als Näherung für das Umfragekollektiv verwendet [5, 6]. Die Datenveröffentlichung erfolgt mit Zustimmung des Deutschen Rheuma-Forschungszentrums (DRFZ).

### Fragebogen

Der verwendete Fragebogen war in fünf Domänen gegliedert (Symptome, Bewegungslevel, aktuelle App-Nutzung, Inhalte einer „Morbus-Bechterew-App“, Nutzung einer „Morbus-Bechterew-App“) (siehe Online-Zusatzmaterial). Es wurden offene Fragen, Fragen mit Einfachantwort, Fragen mit Mehrfachantwort und skalierte Fragen verwendet.

### Datenaufarbeitung und Statistik

Die Daten wurden mittels Excel und GraphPad Prism (Version 5) ausgewertet. Die grafische Darstellung erfolgte mit PowerPoint. In der Subgruppenanalyse wurden die Gruppendaten auf Normalverteilung mittels Shapiro-Wilk-Test getestet. Da keine Normalverteilung ermittelt werden konnte, wurden der Median und der Interquartilsabstand bestimmt. Zeigte sich zwischen zwei Subgruppen ein Unterschied im Median, erfolgte als Signifikanztest der Mann-Whitney-Test. Bei insgesamt 6 durchgeführten Tests erfolgte zur Vermeidung einer α‑Fehler-Kumulation eine Bonferroni-Holm-Korrektur der *p*-Werte mittels eines nicht kommerziellen Onlinetools [7]. Wenn *p* < 0,05 ermittelt wurde, wurden die Unterschiede als signifikant bewertet.

## Ergebnisse

### Erkrankungsausmaß und aktuelle Therapie

Insgesamt nahmen 435 axSpA-Patienten an der Umfrage teil. Von diesen hatten 66,0 % (*n* = 287) nach eigenen Angaben bereits Ankylosierungen. Weiter waren 50,4 % (*n* = 219) der Teilnehmer jünger als 60 Jahre, bei 91,0 % (*n* = 396) war die Erkrankungsdauer länger als 5 Jahre.

Zum Befragungszeitpunkt wurden NSAR oder Coxibe von 50,6 % (*n* = 220) allein oder in Kombination mit anderen Medikamenten eingenommen, 33,6 % (*n* = 146) der Befragten erhielten ein Biologikum. Konventionell-synthetische DMARD wurden von 10,6 % (*n* = 46) und Glukokortikoide von 8,3 % (*n* = 36) eingenommen. Neu zugelassene Januskinase-Inhibitoren (JAK-Inhibitoren)/Upadacitinib wurden bereits von 3 Patienten eingenommen, 21,2 % (*n* = 92) erhielten keine axSpA-spezifische medikamentöse Therapie.

### Abschätzung der Krankheitsaktivität mithilfe der Kerndokumentation und der Proclair-Studie

Auf Basis des Geschlechter- (männlich/weiblich) und Altersverhältnisses (> 60 Jahre/< 60 Jahre) der DVMB-Mitglieder von jeweils annähernd 1:1 wurde eine vergleichbare Stichprobe sowohl aus der Kerndokumentation (*n* = 208) als auch aus der Proclair-Studie (*n* = 1212) gezogen. In der Kerndokumentation lag der mittlere BASDAI für diese Stichprobe bei 3,6 (Standardabweichung [SD] 2,1) und der ASDAS-CRP bei 2,2 (SD 0,9). In der Stichprobe aus der Proclair-Studie lag der mittlere BASDAI bei 4,4 (SD 2,1), der ASDAS-CRP wurde nicht bestimmt. Der mittlere BASDAI und BASFI unterschieden sich bei den Proclair-Patienten nicht relevant zwischen Mitgliedern der DVMB (*n* = 161, mittleres Alter 59 [SD 12] Jahre, mittlerer BASDAI 4,7 [SD 1,9], mittlerer BASFI 4,5 [SD 2,6]) und Nichtmitgliedern (*n* = 1034, mittleres Alter 56 [SD 15] Jahre, mittlerer BASDAI 4,5 [SD 2,1], mittlerer BASFI 4,0 [SD 2,6]).

### Umfang der aktuellen spezifischen Bewegungstherapie

**Figure 1:**
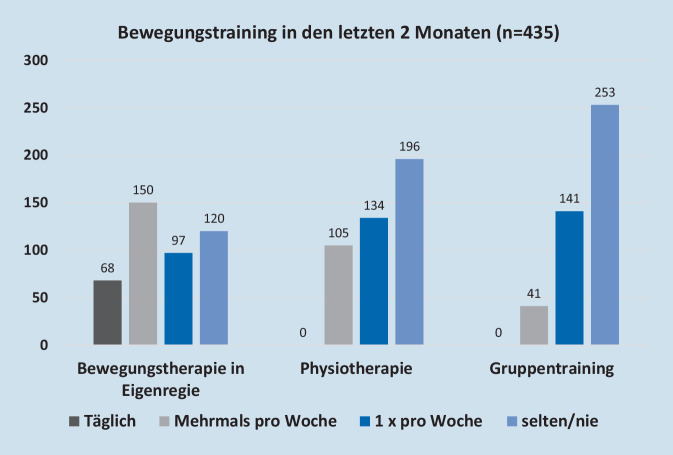

Unter den Teilnehmern trainierten 72,4 % (*n* = 315) mindestens 1 × Woche zu Hause in Eigenregie, während 54,9 % (*n* = 239) mindestens 1 × pro Woche einen Physiotherapeuten aufsuchten und 41,8 % (*n* = 182) mindestens 1 × pro Woche an der Gruppentherapie teilnahmen. Dagegen trainierten 27,6 % (*n* = 120) selten oder nie in Eigenregie, 27,4 % (*n* = 119) trainierten maximal 1 × pro Woche entweder in der Gruppe, zu Hause allein oder beim Physiotherapeuten und 13,6 % (*n* = 59) gaben sogar an, weniger als 1 × pro Woche oder nie zu trainieren (Abb. 1).

### App-Verwendung

**Figure 2:**
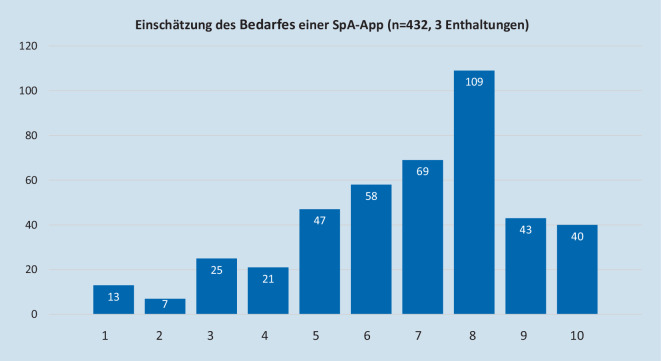

**Figure 3:**
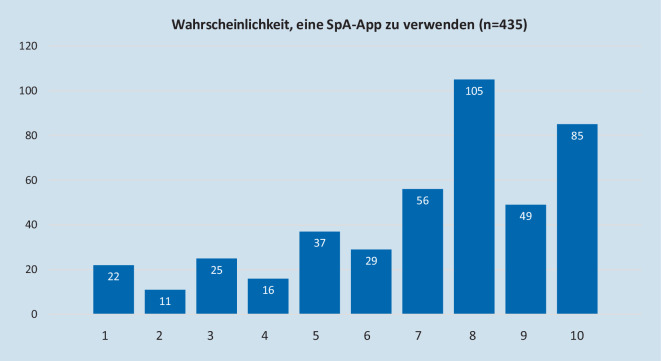

Zum Thema App-Verwendung gaben 83,7 % (*n* = 364) an, dass sie aktuell noch keinerlei App zum Management ihrer Erkrankung verwenden. Von allen Teilnehmern schätzten 84,1 % der Befragten (*n* = 366) auf einer Skala von 1–10 (1 unwichtig, 10 maximal wichtig) ein, dass die Verfügbarkeit einer axSpA-App wichtig wäre (≥ 5 auf der Skala), 44,1 % (*n* = 192) sahen dies sogar als sehr wichtig an (≥ 8) (Abb. 2). Auf die Frage, wie wahrscheinlich die Teilnehmer selbst eine App verwenden würden (auf einer Skala von 1–10), antworteten 83,0 % (*n* = 361) mit wahrscheinlich (≥ 5) und 54,9 % (*n* = 239) sogar mit sehr wahrscheinlich (≥ 8) (Abb. 3). Der Median für den Bedarf einer App lag bei allen Teilnehmern bei 7 von 10, die Wahrscheinlichkeit der App-Nutzung bei 8 von 10.

Teilnehmer unter 60 Jahren schätzten den Bedarf im Median auf 8 von 10 ein und damit höher als die über 60-Jährigen (*p* < 0,001) (Tab. 1). Der Bedarf bei der älteren Gruppe wurde aber nur geringfügig niedriger eingeschätzt, im Median 7 von 10. Die unter 60-Jährigen sahen ebenfalls eine geringfügig höhere Wahrscheinlichkeit, die App selbst zu nutzen (Median 8 von 10, *p* < 0,001) als die Älteren (7 von 10). Zwischen der Gruppe der Biologika/JAK-Inhibitoren-therapierten Patienten und den nicht Biologika/JAK-Inhibitoren-therapierten Patienten gab es weder bei der Frage nach dem Bedarf einer App noch in der Wahrscheinlichkeit der App-Nutzung einen Unterschied, beide schätzten den Bedarf als vorhanden (Median 7) und die Wahrscheinlichkeit der App-Nutzung als sehr hoch ein (Median 8). Bei den unter 60-jährigen Patienten zeigte sich kein Unterschied zwischen Biologika-therapierten und nicht Biologika-therapierten Teilnehmern, beide schätzten den Bedarf und die Nutzungswahrscheinlichkeit sehr hoch ein (jeweils 8 von 10). Beim Vergleich der unter 60-jährigen Biologika/JAK-Inhibitoren-therapierten Patienten mit den über 60-Jährigen, nicht Biologika/JAK-Inhibitoren-therapierten Teilnehmern wurde festgestellt, dass die Jüngeren sowohl den Bedarf (8 vs. 6, *p* < 0,001) als auch die Nutzungswahrscheinlichkeit (8 vs. 7, *p* < 0,001) als höher ansahen. Es wurde kein signifikanter Unterschied zwischen der Gruppe der Patienten, welche häufiger als 1 × pro Woche Bechterew-spezifische Übungen ausführt, und der Gruppe, welche geringer übt, gefunden. Teilnehmer, welche seltener als 1 × pro Woche Rückenschmerzen angaben, sahen den Bedarf genauso hoch an wie Patienten mit häufigeren Schmerzen (beide im Median 7). Die Gruppe mit häufigeren Schmerzen zeigte dafür aber mehr Bereitschaft, die App zu verwenden (8 vs. 7, *p* < 0,001) (Tab. 1). Etwas zurückhaltender fiel die Einschätzung der Teilnehmer aus, ob eine App tatsächlich helfen kann, mit der Krankheit besser zurechtzukommen: Während 31,7 % (*n* = 138) sicher waren, dass eine App ihre Krankheit positiv beeinflussen kann, gaben 59,5 % (*n* = 259) an, dass sich eine App vielleicht positiv auf ihre Krankheitsaktivität auswirken könne. Nur 8,8 % (*n* = 38) gehen nicht davon aus, dass sich eine App positiv auswirke.

**Table 1:** 

	Subgruppe 1			Subgruppe 2		
	*n*	Median	IQR	*n*	Median	IQR
	*Unter 60 Jahre*			*Über 60 Jahre*		
Bedarf einer App	217	8*	3	215	7	3
Wahrscheinlichkeit der Nutzung	219	8*	2	216	7	3
	*Mit Biologikum/JAK-Inhibitoren*			*Ohne Biologikum/JAK-Inhibitoren*		
Bedarf einer App	149	7	3	283	7	3
Wahrscheinlichkeit der Nutzung	149	8	3,5	286	8	4
	*Wenig Bewegung (max. 1* *×* *Wo)*			*Viel Bewegung (>* *1* *×* *Wo)*		
Bedarf einer App	119	7	3	313	7	3
Wahrscheinlichkeit der Nutzung	119	7	4	316	8	3
	*Unter 60 Jahre, Biologikum/JAK-Inhibitoren*			*Über 60 Jahre, ohne Biologikum/JAK-Inhibitoren*		
Bedarf einer App	102	8*	3	168	6	3
Wahrscheinlichkeit der Nutzung	102	8*	3	169	7	3
	*Unter 60 Jahre, Biologikum/JAK-Inhibitoren*			*Unter 60 Jahre, ohne Biologikum/JAK-Inhibitoren*		
Bedarf einer App	102	8	3	115	8	3
Wahrscheinlichkeit der Nutzung	102	8	3	117	8	3
	*Wenig Rückenschmerzen (≤* *1* *×* *Wo)*			*Häufig Rückenschmerzen (>* *1* *×* *Wo)*		
Bedarf einer App	109	7	3	323	7	2
Wahrscheinlichkeit der Nutzung	111	7	4	324	8*	3
	*Unter allen Teilnehmern*					
Bedarf einer App	432	7	3			
Wahrscheinlichkeit der Nutzung	435	8	4			

Gezeigt ist die mediane Einschätzung der unterschiedlichen Subgruppen innerhalb der Befragten axSpA-Patienten auf die Frage nach dem Bedarf und der Nutzungswahrscheinlichkeit einer SpA-App. Die Unterschiede zwischen den jeweiligen zusammengehörenden Gruppen wurden auf Signifikanz getestet

*n* Anzahl, *IQR* Interquartilsabstand, *axSpA* axiale Spondyloarthritis

*p < 0,001

## Diskussion

Bei der axSpA stellt konsequente Bewegungstherapie eine entscheidende therapeutische Säule dar [1]. Art und Umfang der Bewegungsintervention sind aktuell noch Gegenstand der Forschung, auch die deutschsprachige Leitlinie gibt hierzu keine Vorgaben [1]. In randomisiert kontrollierten Studien (RCT), welche den positiven Einfluss der Bewegungstherapie auf Krankheitsparameter bei axSpA zeigten, lag das Mindestmaß an Bewegungstraining bei mehreren Einheiten pro Woche, häufig täglich [2, 8]. In unserer untersuchten Studienpopulation gaben 27 % der Patienten an, lediglich 1 × pro Woche oder seltener axSpA-spezifisch zu trainieren. Erstaunlicherweise trainieren bereits 73 % der Befragten mehrmals pro Woche und 50 % sogar mehrmals pro Woche allein zu Hause axSpA-spezifisch, sodass hier eine Beibehaltung des Trainingsumfanges und individuelle Optimierung wünschenswert wäre. Möglicherweise ist das hohe Maß an Bewegungstherapie dadurch mitbedingt, dass die Umfrage innerhalb einer Selbsthilfegruppe stattfand, in der von einem überdurchschnittlichen Krankheitswissen und hoher Selbstwirksamkeitsüberzeugung auszugehen ist. Der Anteil in der breiten axSpA-Population wird daher wahrscheinlich niedriger liegen. In der Proclair-Studie z. B. wurde bei nur 52 % der 1741 befragten axSpA-Patienten in 2015 Physiotherapie rezeptiert [6]. In beiden Gruppen erscheint der Einsatz von App-basierten Trainingsprogrammen sinnvoll, zum einen um zur Bewegung zu motivieren und zum anderen um die Therapieadhärenz zu fördern. Medizinische Apps haben bereits Einzug in viele Bereich der Medizin gehalten und sind dabei, die Patientenversorgung zu revolutionieren [4]. Teilweise sind diese bereits als DiGA von den Krankenkassen verordnungsfähig, wie zum Beispiel für Depression, Phobien oder Schlaganfallpatienten [4]. Daher ermittelten wir mit dieser Umfrage den Bedarf für die axSpA in einem großen real-world-axSpA-Kollektiv mit 435 Patienten. Da es sich um eine Umfrage unter Laien handelt, konnten keine klinischen Aktivitätsparameter erhoben werden, sodass wir diese aus vergleichbaren Stichproben der Kerndokumentation und Proclair-Studie ableiteten. Hier zeigte sich, dass der ASDAS-CRP eines Referenzkollektivs im Mittel bei 2,2 liegt und damit nach der deutschen Leitlinie eine moderate bis hohe Aktivität in dieser Population anzeigt [1]. Dazu passend liegt der BASDAI in der größeren Proclair-Stichprobe für ein vergleichbares Kollektiv im Mittel bei 4,4, sodass hier ebenfalls von relevanter Krankheitsaktivität ausgegangen werden muss [1]. In den Proclair-Daten zu Krankheitsaktivität und Funktionalität zeigte sich kein relevanter Unterschied zwischen Mitgliedern und Nichtmitgliedern der DVMB. Passend zu diesen erhöhten klinischen Aktivitätsparametern scheint der Bedarf einer solchen App anhand der ermittelten Umfragedaten hoch zu sein, da beinahe 84 % der Befragten sowohl einen Bedarf für eine solche DiGA sehen als auch diese verwenden würden. Obwohl in allen untersuchten Subgruppen eine starke Zustimmung zu einer axSpA-App dokumentiert wurde, zeigten Befragte in bestimmten Subgruppen einen im Verhältnis zu ihrer Kontrollgruppe höheren Bedarf an: Bei Patienten unter 60 Jahren, bei jüngeren Patienten unter Biologika/JAK-Inhibitoren-Therapie und bei Patienten mit häufigen Rückenschmerzen (> 1 × pro Woche) scheint der Bedarf und/oder die Bereitschaft zur Nutzung am höchsten eingeschätzt zu werden. Das aktuelle Pandemieszenario mit mehrmonatigen Lockdowns zeigt uns, dass eine Patientenversorgung mit Physiotherapie bei geschlossenen Physiotherapiepraxen und Therapiegruppen kaum möglich war, sodass hier der Einsatz von DiGA ebenfalls zur Aufrechterhaltung einer optimalen Patientenversorgung sinnvoll wäre. Denkbar ist auch, dass der hohe Zuspruch der Teilnehmer durch deren Erfahrungen mit geschlossenen physiotherapeutischen Einrichtungen und reduziertem Bewegungsangebot während des Lockdowns zustande kommt.

### Limitationen

Die Limitation der Umfrage stellt dar, dass sie innerhalb einer Selbsthilfegruppe stattfand, sodass ein Selektionsbias nicht ausgeschlossen werden kann. Es ist davon auszugehen, dass Mitglieder einer Selbsthilfegruppe ein größeres Krankheitsbewusstsein sowie eine größere Selbstwirksamkeitsüberzeugung haben und damit möglicherweise einen höheren Bedarf sehen als axSpA-Erkrankte der Normalbevölkerung. Denkbar wäre auch der umgekehrte Fall, dass der Bedarf in der Normalbevölkerung höher liegen könnte als unter den gut krankheitsgeschulten Mitgliedern einer Selbsthilfegruppe. Auch wenn der Bedarf der DVMB-Mitglieder mit über 80 % möglicherweise unverhältnismäßig hoch (oder niedrig) im Vergleich zu nicht in einer Selbsthilfegruppe organisierten Patienten angesehen wird, sollte aufgrund dieser hohen Werte auch eine relevante Zustimmung innerhalb der nicht in einer Selbsthilfegruppe organisierten Patienten vorliegen.

### Zusammenfassung

Zusammenfassend scheint es unter den axSpA-Patienten einen großen Bedarf an einer Bewegungs- und Trainings-App zu geben und die Betroffenen scheinen sehr motiviert zu sein, diese in ihre Therapie zu integrieren. Letztlich müssen solche Apps die klinische Testung bestehen und einen Effekt nachweisen, der über die normale Versorgungssituation hinausgeht. Dass ein Bedarf besteht und eine Entwicklung lohnend erscheint, zeigen die Daten dieser Umfrage.

## Fazit für die Praxis

- Bewegungstherapie ist eine der Therapiesäulen bei axialer Spondyloarthritis (axSpA). Eine optimale Bewegungstherapie kann sich positiv auf Funktionalität und Krankheitsaktivität auswirken.
- Aus Sicht von axSpA-Patienten kann eine Bewegungs-App zur Versorgung mit Bewegungstherapie beitragen.
- Über 80 % der Befragten befürworten die Entwicklung einer axSpA-App und würden diese auch selbst nutzen.

## Supplementary Information




