# Supplementary material for: Apps in rheumatology: Is there a need for an app in therapy for axial spondyloarthritis?
Source: Z Rheumatol. 2021 Oct 7;82(3):256–61. [Article in German] doi: 10.1007/s00393-021-01104-1 (PMC10076400; doi:10.1007/s00393-021-01104-1)
Supplement: Supplementary file 1 [file 393_2021_1104_MOESM1_ESM.pdf]

Symptome

1. Wie oft sind diese Symptome in den letzten 2 Monaten bei Ihnen aufgetreten?

	täglich	mehrmals die Woche	ca. 1x pro Woche	seltener	nie
Nacken-, Hüft- oder Rückenschmerzen	◯	◯	◯	◯	◯
Morgensteifigkeit	◯	◯	◯	◯	◯
Ausgeprägte Müdigkeit (Fatigue)	◯	◯	◯	◯	◯
Ein- oder Durchschlafprobleme	◯	◯	◯	◯	◯
sonstige Gelenkschmerzen	◯	◯	◯	◯	◯

2. Wo genau sind die Nacken-, Hüft- oder Rückenschmerzen? (mehrere Antworten möglich)

◯ Nacken

◯ im mittleren/oberen Rücken (Brustwirbelsäule)

◯ Unteren Rücken (Lendenwirbelsäule)

◯ Hüfte (Iliosakralgelenk)

◯ Ich habe keine Nacken-, Hüft- oder Rückenschmerzen

3. Haben Sie Versteifungen (Verknöcherungen) der Wirbelsäule, die Ihre Beweglichkeit einschränken?

◯ Ja

◯ Nein

Bewegungslevel

4. Wie oft haben Sie in den letzten 2 Monaten aktiv Bewegung betrieben?

	täglich	mehrmals die Woche	ca. 1x pro Woche	seltener/nie
Zu Hause durchgeführte Übungen für Morbus Bechterew	○	○	○	○
Physiotherapie/Krankengymnastik mit Therapeuten	○	○	○	○
Bewegungstherapie in der Gruppe	○	○	○	○
leichte Bewegung (z.B. längerer Spaziergang)	○	○	○	○
anderer Sport (z.B. Radfahren, Schwimmen, Kraftsport, ...)	○	○	○	○
Sonstiges (Gymnastik, Yoga, ...)	○	○	○	○

5. Wie viele Minuten in der Woche betreiben Sie aktiv Bewegung? (insgesamt)

○ mehr als 180 Minuten

○ 120-180 Minuten

○ 90-120 Minuten

○ 60-90 Minuten

○ 30-60 Minuten

○ weniger als 30 Minuten

6. Haben Sie das Gefühl, dass Ihnen Bewegung hilft?

◯ Ja

◯ Manchmal

◯ Nein

◯ Weiß nicht

7. Haben Sie das Gefühl, ausreichend Morbus-Bechterew-spezifische Übungen zu kennen?

◯ Ja

◯ Nein

8. Würden Sie sich gerne mehr bewegen?

◯ Ja

◯ Nein

9. OPTIONAL: Wie könnte eine App Sie dabei unterstützen, sich mehr zu bewegen?

Aktuelle App-Nutzung

10. Nutzen Sie bereits Apps, die Ihnen beim Umgang mit Morbus Bechterew helfen? (Mehrfachauswahl möglich)

- ◯ Ja, um spezielle Übungen zu finden
- ◯ Ja, für sonstigen Sport (z.B. Samsung Health, Runtastic, ...)
- ◯ Ja, für die Dokumentation der Medikamenteneinnahme
- ◯ Nein

11. Falls ja, verraten Sie uns, welche App Sie nutzen?

12. Nutzen Sie eine Smartwatch? (z.B. Fitbit, Apple Watch, ...)

- ◯ Ja
- ◯ Nein

Inhalte einer "Morbus-Bechterew-App"

13. Können Sie sich vorstellen, dass eine App Ihnen helfen kann, mit der Krankheit besser zurechtzukommen?

- ◯ Ja
- ◯ vielleicht
- ◯ Nein

14. OPTIONAL: Bei welcher Herausforderung rund um Morbus Bechterew könnte Ihnen eine App helfen?

15. OPTIONAL: Wie könnte eine solche Hilfe aussehen?

16. Können Sie sich vorstellen, regelmäßig unter Anleitung einer Morbus-Bechterew-App ein (Bewegungs-)Übungsprogramm zu absolvieren?

- ◯ Ja
- ◯ vielleicht
- ◯ Nein
- ◯ Weiß nicht

17. Welche Inhalte einer App empfinden Sie als wichtig?

(Vorschläge für mögliche Inhalte einer App. Diese werden hier nicht veröffentlicht.)

18. OPTIONAL: Was würden Sie sich noch von der App wünschen?

19. Wie hoch schätzen Sie den Bedarf für eine Morbus-Bechterew-App ein?

	1	2	3	4	5	6	7	8	9	10	
kein Bedarf	◯	◯	◯	◯	◯	◯	◯	◯	◯	◯	sehr hoher Bedarf

Nutzung einer „Morbus-Bechterew-App“

Die App soll nach höchsten wissenschaftlichen Standards erstellt werden und soll Patienten mit Morbus Bechterew als langfristiger Begleiter dienen. Als geprüftes Medizinprodukt wird Sie größte Datenschutzanforderungen erfüllen und von den gesetzlichen Krankenkassen erstattet werden können. Hierfür ist es nötig, die App beim Arzt oder bei der Krankenkasse zu beantragen.

20. Wie wahrscheinlich ist es, dass Sie eine solche App nutzen würden? (1 = sehr unwahrscheinlich; 10 = sehr wahrscheinlich)

1	2	3	4	5	6	7	8	9	10
○	○	○	○	○	○	○	○	○	○

21. Falls zutreffend: Warum haben Sie Bedenken, eine solche App zu nutzen (z.B. Datenschutz)?

22. (FEEDBACKFRAGE, Testgruppe, wird nicht veröffentlicht)

23. Wie alt sind Sie?

○ 18-29

○ 30-39

○ 40-49

○ 50-59

○ 60-69

○ 70 oder älter

24. Wie lange wissen Sie schon von Ihrer Krankheit?

◯ seit weniger als einem Jahr

◯ seit mehr als 1 Jahr

◯ seit mehr als 5 Jahren

◯ seit mehr als 10 Jahren

25. Nehmen Sie Medikamente ein, und wenn ja welche? (mehrere Antworten möglich)

◯ NSAR (z.B. Ibuprofen, Diclofenac, ...)

◯ Biologika (z.B. TNF-alpha-Hemmer, ...)

◯ Cortisonpräparat

◯ Methotrexat oder Sulfasalazin

◯ Ich nehme keine Medikamente ein.

◯ Sonstiges:
[FREITEXTANTWORT]

26. Gibt es sonst noch etwas, was Sie uns mitteilen wollen?
